# Cross-serotype immunity elicited by a consensus dengue NS1 mRNA vaccine in mice

**DOI:** 10.1371/journal.pone.0355389

**Published:** 2026-08-06

**Authors:** Kittipan Tharakhet, Eakachai Prompetchara, Chirayus Khawsang, Supichcha Saithong, Papatsara Kaewpang, Nongnaphat Yostrerat, Pachara Wangsoontorn, Jenjira Sontikun, Sawaros Tantratorn, Thidarat Khotprathum, Kieu Lam, James Heyes, Drew Weissman, Kiat Ruxrungtham, Chutitorn Ketloy

**Affiliations:** 1 Medical Sciences of Medicine, Faculty of Medicine, Chulalongkorn University, Bangkok, Thailand; 2 Center of Excellence in Vaccine Research and Development (Chula VRC), Faculty of Medicine, Chulalongkorn University, Bangkok, Thailand; 3 Department of Laboratory Medicine, Faculty of Medicine, Chulalongkorn University, Bangkok, Thailand; 4 Genevant Sciences Corporation, Vancouver, Canada; 5 Division of Infectious Diseases, University of Pennsylvania Perelman School of Medicine, Philadelphia, Pennsylvania, United States of America; 6 School of Global Health, Faculty of Medicine, Chulalongkorn University, Bangkok, Thailand; Universidad Cooperativa de Colombia, COLOMBIA

## Abstract

Dengue virus (DENV) remains a major global health burden, with four antigenically distinct serotypes (DENV-1–4) posing a significant challenge for vaccine development. Dengue non-structural protein 1 (NS1) has been associated with additional protection and reduced disease severity, supporting its inclusion in vaccine design. In this study, we designed a consensus NS1 (cNS1) antigen by integrating sequence elements from all four DENV serotypes (78–89% amino acid identity) to enhance cross-serotype antigenic coverage. The cNS1 sequence was encoded as a nucleoside-modified mRNA and formulated in lipid nanoparticles (mRNA–LNPs). Immunization of BALB/c mice with a low dose (0.2 µg) of cNS1 mRNA–LNP induced broadly reactive NS1-specific IgG responses that recognized NS1 proteins from all four serotypes. In addition, the vaccine elicited interferon-γ (IFN-γ)–producing T cell responses against peptide pools derived from multiple DENV serotypes, indicating the activation of cross-reactive cellular immunity. While broad immune recognition was achieved, this was accompanied by lower serotype-specific response magnitudes as a trade-off. In conclusion, the cNS1 mRNA vaccine induces cross-serotype humoral and cellular immune responses in mice, highlighting the potential of consensus antigen design to broaden immune recognition of DENV NS1. These findings support the further development of NS1-based immunogens as complementary components of next-generation dengue vaccines aimed at achieving broad and effective protection.

## Introduction

Dengue virus (DENV), a mosquito-borne flavivirus transmitted primarily by *Aedes aegypti*, remains a major global public health concern. The viral genome comprises a positive-sense, single-stranded RNA genome of approximately 11 kb that encodes a single polyprotein, which is subsequently cleaved into three structural proteins—capsid (C), premembrane/membrane (prM/M), and envelope (E)—and seven nonstructural proteins, including nonstructural protein 1 (NS1) [1,2]. DENV exists as four antigenically distinct serotypes (DENV-1–4), which co-circulate in endemic regions and are associated with a wide spectrum of clinical outcomes, ranging from asymptomatic infection to severe manifestations such as dengue hemorrhagic fever (DHF) and dengue shock syndrome (DSS) [3]. It is estimated that 390 million infections occur annually, placing nearly half of the global population at risk [4,5].

Despite extensive efforts, the development of vaccines capable of eliciting balanced and durable protection against all four dengue virus serotypes remains challenging. Currently licensed tetravalent dengue vaccines have demonstrated that Qdenga addresses key limitations of Dengvaxia by providing protection across serostatus and reducing the risk of severe disease through the inclusion of dengue non-structural antigens, including NS1; however, its remaining limitations in durability, serotype balance, and protection against infection indicate that next-generation vaccines must achieve more balanced, long-lasting, and mechanistically broader immunity. Variability in immunogenicity and efficacy across serotypes and populations highlights the complexity of dengue immunity and the need for complementary strategies that broaden antigenic targets [6–9].

NS1 has emerged as a promising candidate for dengue vaccine development. NS1 is a conserved glycoprotein that is secreted by infected cells and circulates in the bloodstream during acute infection. In addition to its role in viral replication, NS1 contributes to dengue pathogenesis by disrupting endothelial barrier integrity and triggering inflammatory responses [10–12]. Antibodies against NS1 have been shown to neutralize its pathogenic effects, including NS1-induced endothelial dysfunction, and to reduce disease severity in animal models [13–15]. Notably, NS1 has been demonstrated to induce endothelial permeability and vascular leakage, effects that can be prevented by NS1-targeted immunization [13]. These findings suggest that NS1-directed immunity may complement conventional approaches focused on structural antigens.

Given the genetic diversity among DENV serotypes, consensus antigen design represents a potential strategy to enhance cross-serotype immune recognition. Consensus sequences are generated by integrating genetic information from multiple viral strains to produce representative antigens enriched for conserved regions. This strategy has been applied to enhance the breadth of immune responses against several viral pathogens [16,17], although its application to DENV NS1 remains limited.

The mRNA vaccine platform offers a flexible and scalable approach for antigen delivery. Nucleoside-modified mRNA formulated in lipid nanoparticles (LNPs) enables efficient *in vivo* antigen expression and induces both humoral and cellular immune responses. The rapid development and deployment of mRNA vaccines during the COVID-19 pandemic demonstrated the utility of this platform for emerging infectious diseases [18–20]. In dengue research, mRNA-based vaccines encoding viral antigens have shown promising immunogenicity and protective efficacy in preclinical models [21,22].

Importantly, unlike structural proteins that predominantly induce neutralizing antibodies, NS1 confers protection through mechanisms independent of viral neutralization. NS1-specific antibodies can recognize infected cells and mitigate NS1-mediated endothelial dysfunction, a key driver of dengue pathogenesis [13]. Consistent with this, an mRNA–LNP–encoded NS1 vaccine has been shown to confer protection against DENV-2 challenge in mice, primarily by reducing disease severity rather than preventing infection [22]. These findings support the inclusion of NS1 as a complementary antigen to enhance vaccine efficacy by targeting both viral replication and disease-associated host responses.

In this study, we designed a consensus NS1 (cNS1) antigen based on sequence data from DENV serotypes 1–4 to enhance antigenic coverage. The cNS1 antigen was encoded as a nucleoside-modified mRNA and formulated in LNPs. We evaluated antigen expression, immunogenicity, and the breadth of humoral and cellular immune responses in a murine model. Our findings demonstrate that cNS1 mRNA vaccination induces cross-reactive immune responses, supporting the potential utility of a consensus NS1 design as a component of next-generation dengue vaccines.

## Materials and methods

All animal procedures were conducted in accordance with the Ethical Principles and Guidelines for the Use of Animals for Scientific Purposes. The study protocol was approved by the Institutional Animal Care and Use Committee (IACUC) of the Faculty of Medicine, Chulalongkorn University (approval number: 2491012). All experiments were performed in accordance with relevant institutional guidelines and regulations for animal care and use.

### Cell culture

African green monkey kidney cells (Vero, ATCC CCL-81) were obtained from ATCC (Manassas, VA, USA). Cells were maintained in Minimum Essential Medium (MEM) supplemented with 10% heat-inactivated fetal bovine serum (FBS), L-glutamine, and penicillin–streptomycin (Gibco, USA). Cultures were incubated at 37 °C in a humidified atmosphere containing 5% CO_2_.

### Consensus NS1 design

**Fig 1:**
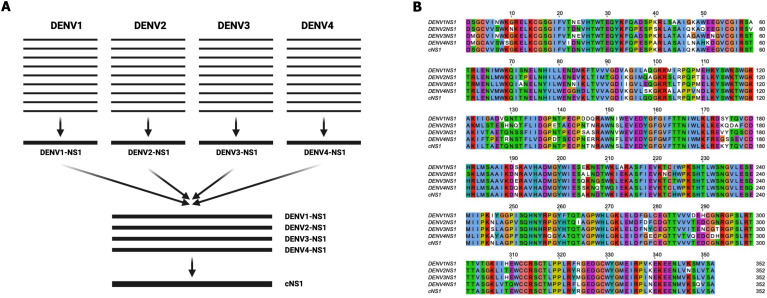
Design and sequence conservation of the consensus NS1 antigen. (A) Workflow for the design of the cNS1 antigen. Twenty-five representative NS1-containing polyprotein sequences from each dengue virus serotype (DENV-1–4), selected from geographically and temporally diverse isolates, were aligned to generate serotype-specific consensus sequences (DENV1-NS1, DENV2-NS1, DENV3-NS1, and DENV4-NS1). These four consensus sequences were subsequently aligned to generate the final consensus NS1 (cNS1) sequence. (B) Multiple sequence alignment of NS1 proteins from DENV-1–4 and the cNS1 sequence. The alignment is presented in segments for clarity, with amino acid residues colored according to the ClustalX scheme to indicate sequence conservation.

**Fig 2:**
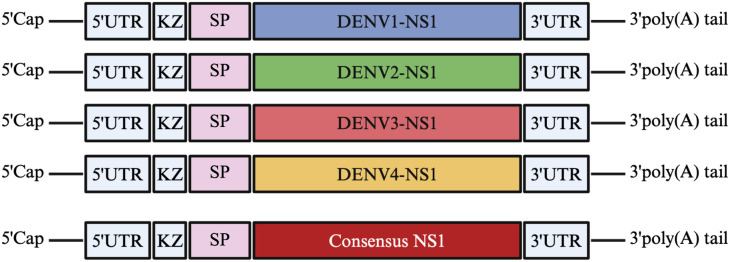
Schematic representation of serotype-specific NS1 and cNS1 mRNA constructs. Diagram of nucleoside-modified mRNA constructs encoding dengue virus serotype-specific NS1 proteins (DENV-1–4) and cNS1. Each construct contains a 5′ cap structure, 5′ untranslated region (UTR), Kozak sequence, signal peptide (SP), NS1 coding sequence, 3′ UTR, and a genetically encoded poly(A) tail consisting of 101 adenine residues. The *in vitro*–transcribed mRNAs were subsequently formulated in lipid nanoparticles (LNPs) for downstream experiments.

NS1 protein sequences from all four dengue virus serotypes (DENV-1–4) were retrieved from the NCBI Protein database. To generate representative serotype-specific consensus sequences while maintaining a manageable dataset for sequence analysis, 25 representative NS1-containing polyprotein sequences per serotype, collected between 1964 and 2022, were selected. The selected sequences represented geographically and temporally diverse isolates from multiple countries (S1 Table). Sequences containing incomplete coding regions, ambiguous amino acid residues, duplicated entries, or extensive gaps were excluded from further analysis. Multiple sequence alignments were performed using ClustalW (version 2.1) to generate serotype-specific consensus sequences, designated as DENV1-NS1, DENV2-NS1, DENV3-NS1, and DENV4-NS1 (Fig 1A). Subsequently, the four serotype-specific consensus sequences were aligned to generate the final cNS1 sequence (Fig. 1B). This two-step consensus design strategy was intended to ensure balanced representation of all four dengue virus serotypes while preserving conserved amino acid residues shared across DENV-1–4. Two commonly used signal peptides (SPs), tissue plasminogen activator (tPA) and immunoglobulin E (IgE), were incorporated upstream of the NS1 coding sequence in all constructs (Fig 2). Synthetic genes encoding the codon-optimized cNS1 and serotype-specific NS1 sequences were designed with codon optimization for human expression and synthesized by GenScript (Piscataway, NJ, USA) and GenScript Biotech (Singapore).

### *In vitro* transcription and mRNA encapsulation

Plasmid DNA encoding serotype-specific dengue virus NS1 or the cNS1 sequence was subcloned into the pUC-ccTEV-A101 vector for *in vitro* transcription. The pUC-ccTEV-A101 vector incorporates proprietary 5′ and 3′ untranslated regions (UTRs) optimized for mRNA stability and translation efficiency. All vaccine constructs were generated using the same mRNA backbone and regulatory elements. Linearized plasmids were used as templates for mRNA synthesis using the MEGAscript™ T7 Transcription Kit (Invitrogen, USA) according to the manufacturer’s instructions. During transcription, uridine was fully substituted with 1-methylpseudouridine-5′-triphosphate (m¹Ψ) (TriLink, USA) to enhance mRNA stability and reduce innate immune activation. Co-transcriptional capping was performed using CleanCap® AG (3′-O-Me) (TriLink, USA), and all mRNA constructs contained a genetically encoded poly(A) tail consisting of 101 adenine residues. Following transcription, mRNA was purified using a cellulose-based purification method to remove double-stranded RNA (dsRNA) contaminants. RNA integrity was assessed by agarose gel electrophoresis, and residual dsRNA was evaluated by dot blot analysis following cellulose-based purification (S1 Fig).

Purified mRNA was encapsulated in LNPs using a self-assembly process comprising an ionizable lipid (3D-P-DMA), DSPC, cholesterol, and PEG2000-C-DMA at a molar ratio of 50:10:38.5:1.5, respectively. Following encapsulation, the mRNA-LNP formulations were subjected to buffer exchange by dialysis into the final formulation buffer prior to physicochemical characterization, including measurement of particle size (Z-average), polydispersity index (PDI), and encapsulation efficiency (S2 Table). The resulting mRNA–LNP formulations were stored at −80 °C until use.

### *In vitro* mRNA transfection and protein expression analysis

Vero cells were transfected with mRNA using Lipofectamine™ MessengerMAX™ (Thermo Fisher Scientific, USA) according to the manufacturer’s instructions. Briefly, 2.5 µg of mRNA was diluted in Opti-MEM™ medium and combined with the transfection reagent prior to addition to cells seeded in 6-well plates. Cells were incubated for 24 h before downstream analyses.

For immunofluorescence staining, transfected cells cultured on coverslips were fixed with cold acetone and incubated with a rabbit anti-DENV2-NS1 polyclonal antibody (Invitrogen, USA; catalog no. PA5-32207), followed by fluorophore-conjugated secondary antibodies. Nuclei were counterstained with DAPI. Images were acquired using a confocal fluorescence microscope.

For detection of secreted NS1, culture supernatants were collected and separated by SDS–PAGE, followed by transfer onto nitrocellulose membranes. Membranes were blocked with 5% skim milk in Tris-buffered saline with Tween 20 (TBST) and incubated with the same anti-NS1 primary antibody, followed by horseradish peroxidase (HRP)–conjugated secondary antibodies. Protein bands were visualized using an enhanced chemiluminescence detection system.

### Immunization study in BALB/c mice

Six- to eight-week-old female BALB/c mice were purchased from Nomura Siam International (Bangkok, Thailand). Mice were randomly assigned to six groups (n = 5 per group) and immunized intramuscularly with mRNA–LNP formulations at a dose of 0.2 µg per mouse using a prime–boost regimen on days 0 and 21. Experimental groups included serotype-specific NS1 mRNA vaccines, a cNS1 mRNA vaccine, and a normal saline solution (NSS) control.

**Fig 3:**
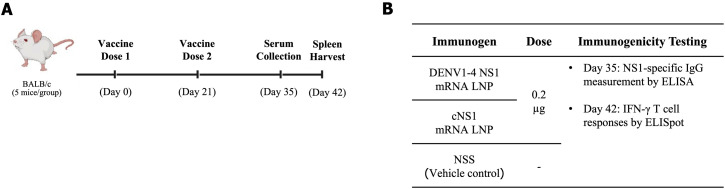
Immunization strategy for evaluation of NS1 mRNA vaccines in BALB/c mice. (A) Schematic representation of the immunization schedule. BALB/c mice (n = 5 per group) were immunized intramuscularly with NS1 mRNA–LNP vaccines using a prime–boost regimen on days 0 and 21. Serum samples were collected on day 35 for antibody analysis, and mice were euthanized on day 42 for splenocyte isolation. (B) Experimental groups included mRNA vaccines encoding serotype-specific NS1 proteins (DENV-1–4), cNS1, and normal saline solution (NSS) as a negative control. Immunogenicity was evaluated by ELISA for NS1-specific IgG and IFN-γ ELISpot for antigen-specific T cell responses.

Blood samples were collected from the submandibular vein on day 35 for antibody analysis. Mice were euthanized three weeks after the booster immunization (day 42), and spleens were harvested for the evaluation of cellular immune responses. A schematic overview of the immunization schedule and experimental groups is presented in Fig 3A and 3B.

### Enzyme-linked immunosorbent assay (ELISA)

Sera collected from mice immunized with either the serotype-specific NS1 mRNA vaccines or the cNS1 mRNA vaccine were evaluated against each recombinant serotype-specific NS1 protein to assess both serotype-specific and cross-serotype antibody reactivity. NS1-specific IgG antibodies were quantified by ELISA using commercially available recombinant dengue virus NS1 proteins representing serotypes 1–4 (Sino Biological, China) as coating antigens. Ninety-six-well plates were coated overnight at 4 °C with each recombinant NS1 protein (0.1 µg/well). Plates were blocked with 1% bovine serum albumin (BSA) in phosphate-buffered saline containing 0.05% Tween 20 (PBST). Serially diluted mouse serum samples were added and incubated, followed by washing and incubation with horseradish peroxidase (HRP)–conjugated goat anti-mouse IgG. Color development was performed using 3,3′,5,5′-tetramethylbenzidine (TMB). Absorbance was measured at 450 nm using a VICTOR Nivo™ multimode plate reader (PerkinElmer, USA). Endpoint titers were determined using four-parameter logistic (4PL) regression analysis in GraphPad Prism (version 10; GraphPad Software, USA), and midpoint titers were calculated by interpolation from the fitted curves.

### Mouse IFN-γ enzyme-linked immunospot (ELISpot) assay

Cellular immune responses were evaluated using a mouse IFN-γ ELISpot assay (Mabtech, Sweden) according to the manufacturer’s instructions. Splenocytes were isolated from immunized mice and seeded at 5 × 10^5^ cells per well in pre-coated ELISpot plates. Cells were stimulated with overlapping peptide pools spanning dengue virus NS1 proteins. Peptides corresponding to DENV-2, DENV-3, and DENV-4 NS1 were obtained from BEI Resources (NIAID, NIH; cat. nos. NR-508, NR-2753, and NR-2755, respectively). DENV1-NS1 peptide pools were synthesized commercially by Mimotopes Pty Ltd (Victoria, Australia). After incubation for 40 h at 37 °C in a 5% CO_2_ atmosphere, plates were incubated with a biotinylated anti-IFN-γ detection antibody, followed by streptavidin–alkaline phosphatase. Spots were developed using BCIP/NBT substrate. Spot-forming cells (SFCs) were enumerated using an ImmunoSpot® analyzer, and results were expressed as SFCs per 10^6^ splenocytes after subtraction of background counts from unstimulated control wells.

### Statistical analysis

Statistical analyses were performed using GraphPad Prism (version 10; GraphPad Software, USA). Comparisons between experimental groups were performed using the two-tailed Mann–Whitney test. Antibody responses are presented as geometric mean titers (GMTs) with geometric standard deviation (SD), whereas ELISpot data are expressed as mean ± SD. A p value < 0.05 was considered statistically significant.

## Results

### Design and generation of a consensus NS1 mRNA construct

A cNS1 sequence was generated to represent conserved regions across dengue virus serotypes 1–4. NS1 protein sequences (352 amino acids) from geographically and temporally diverse isolates (1964–2022; 25 sequences per serotype) were aligned to generate serotype-specific consensus sequences, which were subsequently combined to produce a cNS1 (Fig 1A). Previous studies have reported that NS1 proteins share approximately 68.5–79.5% amino acid identity among dengue virus serotypes [23], whereas the resulting cNS1 sequence exhibited higher identity to the individual serotypes (78.7–88.9%; Table 1). All NS1 sequences retained the canonical 352–amino acid length without insertions or deletions, and conserved structural regions were preserved in the consensus sequence (Fig 1B). Key structural features, including conserved domains and N-linked glycosylation sites (N130 and N207), were preserved.

**Table 1:** Pairwise amino acid identity between cNS1 and serotype-specific dengue virus NS1 consensus sequences.

Comparison	Identity^a^ (%)
cNS1 vs. DENV-1 consensus	82.7
cNS1 vs. DENV-2 consensus	88.9
cNS1 vs. DENV-3 consensus	84.9
cNS1 vs. DENV-4 consensus	78.7

^a^Pairwise sequence identity was calculated from multiple sequence alignments generated using ClustalW (version 2.1).

All NS1 sequences were human codon-optimized and incorporated into nucleoside-modified mRNA constructs containing regulatory elements for efficient expression and formulated in LNPs for downstream applications. A schematic overview of the DENV-1–4 NS1 and cNS1 mRNA constructs is shown in Fig 2.

### Different signal peptides support NS1 expression and secretion

**Fig 4:**
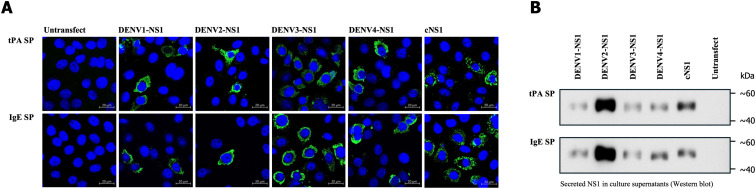
Expression and secretion of serotype-specific NS1 and cNS1 with different signal peptides. A) Immunofluorescence analysis of NS1 expression in transfected cells. Cells were transfected with mRNA constructs encoding DENV-1–4 NS1 or cNS1 containing either the tissue plasminogen activator (tPA) or immunoglobulin E (IgE) signal peptide. NS1 expression (green) was detected using a DENV2-NS1-specific polyclonal antibody (Invitrogen, USA; catalog no. PA5-32207), and nuclei were counterstained with DAPI (blue). Untransfected cells served as negative controls. (B) Western blot analysis of secreted NS1 proteins. Culture supernatants from transfected cells were analyzed using the same anti-NS1 antibody.

To evaluate the impact of signal peptide selection on NS1 expression and secretion, tPA and IgE signal peptides were fused upstream of the NS1 coding sequence. Immunofluorescence analysis demonstrated robust NS1 expression in cells transfected with both tPA- and IgE-containing constructs, with a predominantly perinuclear and reticular staining pattern consistent with endoplasmic reticulum localization, whereas no signal was detected in untransfected controls (Fig 4A). Western blot analysis of culture supernatants confirmed that both tPA and IgE signal peptides supported NS1 secretion (Fig 4B). However, differences in band intensity were observed among serotypes, with DENV2-NS1 showing the strongest signal, whereas other serotypes exhibited lower signal intensities. This may be partly attributed to antibody recognition bias, as detection was performed using a rabbit anti-DENV2-NS1 polyclonal antibody, which may preferentially recognize epitopes conserved in DENV-2, thereby limiting direct quantitative comparison of protein abundance between constructs. Despite these differences, both signal peptides supported efficient NS1 expression and secretion. Based on the overall performance observed, the IgE signal peptide was selected for subsequent studies, consistent with its use in previous mRNA vaccine studies.

In silico prediction using SignalP 6.0 indicated high signal peptide functionality for both sequences (probability > 0.97), with predicted cleavage sites at positions 18–19 for IgE and 22–23 for tPA (S2 Fig). Consistent with these findings, both signal peptides supported efficient NS1 expression and secretion *in vitro*.

### Consensus NS1 mRNA vaccine induces balanced cross-reactive antibody responses across all four dengue virus serotypes

NS1-specific IgG levels were measured in sera collected two weeks after the booster immunization (day 35) by ELISA using recombinant NS1 proteins from dengue virus serotypes 1–4.

**Fig 5:**
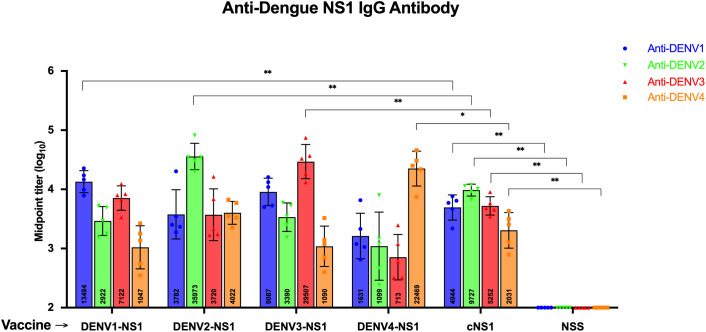
DENV NS1-specific IgG antibody responses analyzed by ELISA. Sera collected from mice two weeks after the second immunization (day 35) were assessed against recombinant NS1 proteins from dengue virus serotypes 1–4. Antibody titers are presented as midpoint titers (log_10_). Each group includes mice immunized with serotype-specific NS1 mRNA vaccines (DENV-1–4), cNS1, or normal saline solution (NSS) as a negative control. Data are shown as GMT ± SD, with individual data points representing each mouse (n = 5 per group). Statistical significance between groups was determined using the Mann–Whitney test (*p < 0.05, **p < 0.01).

Serotype-specific NS1 mRNA vaccines induced strong homologous responses, with the highest antibody levels observed against their corresponding antigens, whereas reactivity to heterologous NS1 proteins was lower (Fig 5). GMTs for homologous responses were 13,494 for DENV-1, 35,973 for DENV-2, 29,507 for DENV-3, and 22,469 for DENV-4. In contrast, the cNS1 vaccine elicited IgG responses against all four serotypes, with GMTs of 4,944, 9,727, 5,252, and 2,031 for DENV-1–4, respectively. Although lower than homologous responses, these antibodies exhibited a more balanced cross-reactive profile. No NS1-specific antibodies were detected in the normal saline control group. These results demonstrate that cNS1 mRNA vaccination induces broadly cross-reactive NS1-specific antibody responses.

### Consensus NS1 mRNA vaccine induces broad IFN-γ T cell responses

IFN-γ–secreting cells were measured in splenocytes collected three weeks after the booster immunization (day 42) using pooled overlapping peptide libraries spanning the NS1 proteins of dengue virus serotypes 1–4.

**Fig 6:**
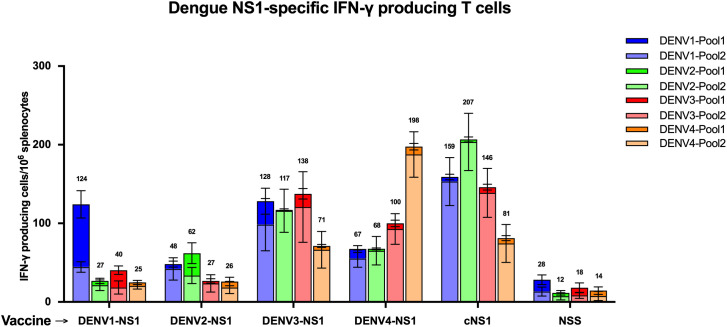
DENV NS1-specific T cell responses analyzed by ELISpot. Splenocytes collected from mice three weeks after the booster immunization (day 42) were stimulated with pooled overlapping peptide libraries representing NS1 proteins from dengue virus serotypes 1–4. Responses are presented as IFN-γ–producing spot-forming cells (SFCs) per 10^6^ splenocytes. Data are shown as mean ± SD.

Serotype-specific NS1 mRNA vaccines induced detectable IFN-γ–producing T cell responses, which were higher with homologous than heterologous peptide stimulation (Fig 6). In contrast, the cNS1 vaccine elicited IFN-γ responses across all four serotypes, with 159, 207, 146, and 81 spot-forming cells per 10^6^ splenocytes for DENV-1–4, respectively. Responses in the cNS1 group were broadly distributed, whereas serotype-specific vaccines were biased toward individual peptide pools. Minimal background responses were observed in the NSS control group. These results demonstrate that cNS1 induces broad, cross-reactive cellular immune responses.

## Discussion

In this study, we demonstrate that an mRNA-encoding consensus NS1 (cNS1) vaccine induces broadly cross-reactive humoral and cellular immune responses across all four dengue virus serotypes. In contrast to serotype-specific NS1 vaccines, which elicited strong homologous but limited heterologous responses, cNS1 generated more balanced IgG responses and IFN-γ–producing T cell responses spanning DENV-1–4. These findings support the potential of consensus antigen design to enhance the breadth of dengue vaccine–induced immunity.

The enhanced cross-reactivity observed with cNS1 is likely driven by its consensus-based sequence, which incorporates conserved regions across dengue virus serotypes. This design may facilitate the presentation of shared epitopes to both B and T cells, thereby promoting cross-serotype immune recognition. While antibody responses induced by cNS1 were lower than homologous responses induced by serotype-specific vaccines, they were more evenly distributed across serotypes, suggesting a trade-off between magnitude and breadth of humoral immunity. Similarly, cNS1 elicited IFN-γ–producing T cell responses against all serotypes, although variation in response magnitude was observed, which may reflect epitope immunodominance and MHC-restricted antigen presentation in the BALB/c model. Notably, the enhanced breadth of immune responses induced by cNS1 was accompanied by a reduced response magnitude against certain serotypes, most prominently DENV-4. This observation is particularly relevant given that DENV-4 is often less immunogenic in both natural infection and vaccination settings and has been associated with comparatively lower vaccine efficacy in some studies, highlighting an important trade-off between breadth and peak serotype-specific immunogenicity.

Previous studies have shown that NS1-based vaccines can confer protection through mechanisms distinct from neutralizing antibodies, including the inhibition of NS1-mediated endothelial dysfunction and vascular leakage [10,11,24]. In addition, because NS1 is not a structural component of the virion, NS1-specific antibodies are unlikely to mediate antibody-dependent enhancement (ADE), a major safety concern in dengue vaccine development [2]. However, most NS1 vaccine candidates reported to date have focused on single-serotype antigens, particularly DENV-2, which may limit cross-protective efficacy against genetically diverse dengue virus strains [22,25]. Our findings extend these observations by demonstrating that a consensus NS1 antigen can broaden both antibody and T cell responses across serotypes.

Clinical observations further support the relevance of NS1-directed immunity. The live-attenuated tetravalent dengue vaccine TAK-003 (Qdenga), which is based on a DENV-2 backbone, has been shown to induce both NS1-specific humoral and cellular immune responses in vaccinated individuals [7,26,27]. Notably, NS1 responses are more prominent against the DENV-2 component, consistent with the vaccine backbone design. In addition, sera from TAK-003 recipients have been reported to inhibit NS1-induced endothelial hyperpermeability *in vitro*, suggesting that anti-NS1 antibodies may contribute to protection by mitigating NS1-mediated vascular leakage [28]. These clinical findings are consistent with our observations that NS1-targeted responses can be broadly induced and may contribute to protective mechanisms beyond virus neutralization.

In addition to antigen design, our results show that both tPA and IgE signal peptides supported efficient NS1 expression and secretion, suggesting that antigen-intrinsic structural features may play an important role in determining secretion efficiency. The preservation of conserved N-linked glycosylation sites (N130 and N207), which are critical for NS1 folding, stability, and extracellular release [25,29], further supports this observation. Retention of these structural elements in a full-length NS1 construct likely facilitates proper antigen conformation and the presentation of conformational epitopes, supporting the observation of robust humoral and cellular immune responses. Notably, the cNS1 mRNA vaccine induced robust immune responses at a low dose (0.2 µg per immunization), suggesting a potential dose-sparing advantage.

Despite these promising findings, several limitations should be acknowledged. First, the study was conducted in a single small-animal model, which may not fully reflect human immune responses. Second, protective efficacy was not directly evaluated in a dengue virus challenge model. Future studies should investigate whether combining the consensus NS1 antigen with structural antigens, such as prME, can provide complementary immune mechanisms by inducing both neutralizing antibody responses and NS1-directed immunity. Third, circulating NS1 protein levels following vaccination and the functional activity of the induced immune responses, particularly their ability to prevent NS1-mediated vascular leakage, were not evaluated. Future studies addressing these aspects will provide a more comprehensive understanding of the mechanisms underlying NS1-directed immunity. Fourth, cellular immune responses were evaluated solely by IFN-γ ELISpot. Although IFN-γ is a well-established indicator of antigen-specific T cell responses, additional characterization of cytokine profiles (e.g., IL-2, TNF-α, IL-4, and IL-10) would provide a more comprehensive understanding of the quality and functional characteristics of vaccine-induced cellular immunity.

In summary, this study demonstrates that a consensus NS1 mRNA vaccine can induce balanced and cross-reactive humoral and cellular immune responses across dengue virus serotypes. These findings support the potential of consensus antigen–based mRNA vaccine design as a promising strategy for improving the breadth of dengue vaccine–induced immunity and warrant further evaluation in advanced preclinical and clinical studies.

## Supporting information

S1 Fig Quality control of in vitro–transcribed mRNAs. (A) Agarose gel electrophoresis demonstrating the integrity of in vitro–transcribed mRNAs encoding DENV-1–4 NS1 and cNS1 constructs before (IVT) and after cellulose-based purification. (B) Residual double-stranded RNA (dsRNA) was evaluated by dot blot analysis using the J2 monoclonal antibody before (IVT) and after cellulose-based purification.
(DOCX)

S2 Fig *In silico* prediction of signal sequences for the cNS1 construct. Signal peptide predictions were performed using SignalP 6.0 with the Sec/SPI model. (A) Predicted signal peptide profile of the IgE signal sequence. (B) Predicted signal peptide profile of the tPA signal sequence. (C) Summary of predicted signal peptide characteristics, including cleavage sites and probability scores.
(DOCX)

S1 Table Representative NS1-containing polyprotein sequences used for consensus NS1 design. Representative NS1-containing polyprotein sequences were retrieved from the NCBI Protein database and selected to maximize geographic and temporal diversity. Sequences containing incomplete coding regions, ambiguous amino acid residues, duplicated entries, or extensive gaps were excluded from the analysis.
(DOCX)

S2 Table Physicochemical characteristics of mRNA-LNP formulations. Particle size is reported as the Z-average diameter measured by dynamic light scattering (DLS). PDI, polydispersity index; EE, encapsulation efficiency.
(DOCX)
