# Supplementary material for: Cross-serotype immunity elicited by a consensus dengue NS1 mRNA vaccine in mice
Source: PLoS One. 2026 Aug 6;21(8):e0355389. doi: 10.1371/journal.pone.0355389 (PMC13446656; doi:10.1371/journal.pone.0355389)
Supplement: Supplementary file 3 [file pone.0355389.s003.docx]

**S1 Table.**

| **Serotype** | **Country** | **Year** | **Accession** |  | **Serotype** | **Country** | **Year** | **Accession** |
| --- | --- | --- | --- | --- | --- | --- | --- | --- |
| DENV-1 | Indonesia | 2012 | AHK09922.1 |  | DENV-3 | Indonesia | 2012 | AHK09945.1 |
| DENV-1 | China | 2014 | AJR21214.1 |  | DENV-3 | Indonesia | 2016 | QBB90023.1 |
| DENV-1 | China | 2018 | QIC53443.1 |  | DENV-3 | Myanmar | 2018 | QTE05719.1 |
| DENV-1 | China | 2019 | QFS19562.1 |  | DENV-3 | China | 2013 | ASN77913.1 |
| DENV-1 | Argentina | 2010 | AHF50492.1 |  | DENV-3 | China | 2019 | ODZ58870.1 |
| DENV-1 | Brazil | 2016 | QNL13565.1 |  | DENV-3 | Australia | 2016 | QNJ99453.1 |
| DENV-1 | Brazil | 2019 | QNL13557.1 |  | DENV-3 | Brazil | 2006 | AEV42062.1 |
| DENV-1 | Haiti | 2014 | ALJ53459.1 |  | DENV-3 | Ecuador | 2001 | UDI89638.1 |
| DENV-1 | Malaysia | 2014 | ARO84722.1 |  | DENV-3 | Sri Lanka | 2020 | UDM48676.1 |
| DENV-1 | USA | 2010 | AFJ91714.1 |  | DENV-3 | Thailand | 1991 | QZE60613.1 |
| DENV-1 | Laos | 2008 | AGT63075.1 |  | DENV-3 | Thailand | 1994 | UDI89637.1 |
| DENV-1 | Thailand | 1964 | QTX92473.1 |  | DENV-3 | Thailand | 2003 | QTX93611.1 |
| DENV-1 | Thailand | 2005 | QOQ57118.1 |  | DENV-3 | Thailand | 2007 | QBK46952.1 |
| DENV-1 | Thailand | 2011 | UNO86144.1 |  | DENV-3 | Thailand | 2016 | ANG57778.1 |
| DENV-1 | Thailand | 2015 | QJF23946.1 |  | DENV-3 | Thailand | 2012 | QFS19536.1 |
| DENV-1 | Thailand | 2019 | QXX05302.1 |  | DENV-3 | Thailand | 2015 | ASS36951.1 |
| DENV-1 | Thailand | 2019 | QXX05303.1 |  | DENV-3 | Thailand | 2018 | USW91935.1 |
| DENV-1 | Thailand | 2019 | QXX05304.1 |  | DENV-3 | India | 2018 | UBI73841.1 |
| DENV-1 | Thailand | 2019 | QXX05305.1 |  | DENV-3 | India | 2022 | UZZ63633.1 |
| DENV-1 | Thailand | 2019 | QXX05306.1 |  | DENV-3 | Nicaragua | 2013 | QUD37256.1 |
| DENV-1 | Cambodia | 2019 | UAV34195.1 |  | DENV-3 | Mexico | 2019 | QPJ75914.1 |
| DENV-1 | Myanmar | 2019 | QTE20483.1 |  | DENV-3 | Kenya | 2015 | QIB99401.1 |
| DENV-1 | Myanmar | 2022 | WAL91252.1 |  | DENV-3 | Burkina Faso | 2017 | QIS48880.1 |
| DENV-1 | Somalia | 2011 | QZL17725.1 |  | DENV-3 | Malaysia | 2014 | QBQ58392.1 |
| DENV-1 | India | 2016 | UBI73853.1 |  | DENV-3 | Singapore | 2016 | QFN25407.1 |
| DENV-2 | Indonesia | 2010 | AHK09926.1 |  | DENV-4 | Thailand | 1994 | UDI89644.1 |
| DENV-2 | Thailand | 1964 | NP_739584.2 |  | DENV-4 | Thailand | 2005 | QTX92397.1 |
| DENV-2 | Thailand | 1999 | QTX93225.1 |  | DENV-4 | Thailand | 2012 | QFS19549.1 |
| DENV-2 | Thailand | 2002 | QTX93158.1 |  | DENV-4 | Thailand | 2013 | ASS36950.1 |
| DENV-2 | Thailand | 2011 | QFS19460.1 |  | DENV-4 | Thailand | 2021 | QTE18638.1 |
| DENV-2 | Thailand | 2011 | QFS19461.1 |  | DENV-4 | Indonesia | 2010 | AHK09949.1 |
| DENV-2 | Thailand | 2011 | QFS19462.1 |  | DENV-4 | Indonesia | 2019 | UDW38833.1 |
| DENV-2 | Thailand | 2011 | QFS19463.1 |  | DENV-4 | Brazil | 2011 | AFX65881.1 |
| DENV-2 | Thailand | 2019 | QXY23100.1 |  | DENV-4 | Brazil | 2012 | UKS89504.1 |
| DENV-2 | Thailand | 2019 | QXY23101.1 |  | DENV-4 | Myanmar | 2018 | QTE05843.1 |
| DENV-2 | Thailand | 2019 | QXY23102.1 |  | DENV-4 | India | 2017 | QOV09272.1 |
| DENV-2 | China | 2014 | AJP06043.1 |  | DENV-4 | India | 2021 | UZI17961.1 |
| DENV-2 | Brazil | 2019 | QNL13566.1 |  | DENV-4 | Cambodia | 2007 | AHG23293.1 |
| DENV-2 | Brazil | 2022 | WAB71368.1 |  | DENV-4 | Cambodia | 2020 | QZP79864.1 |
| DENV-2 | Singapore | 2009 | AEI29060.1 |  | DENV-4 | Paraguay | 2018 | QHZ01382.1 |
| DENV-2 | Sri Lanka | 2017 | QIJ58805.1 |  | DENV-4 | China | 2017 | QPI70488.1 |
| DENV-2 | Malaysia | 2014 | ARO84705.1 |  | DENV-4 | China | 2020 | QPJ20200.1 |
| DENV-2 | Haiti | 2016 | AOE23002.1 |  | DENV-4 | Kenya | 2015 | QIB99408.1 |
| DENV-2 | Venezuela | 2016 | AWI47685.1 |  | DENV-4 | Malaysia | 2014 | QBQ58394.1 |
| DENV-2 | Vietnam | 1995 | AGO67249.1 |  | DENV-4 | Malaysia | 2021 | USW90911.1 |
| DENV-2 | Vietnam | 2022 | WAP37906.1 |  | DENV-4 | Venezuela | 2000 | ACO06199.1 |
| DENV-2 | Cambodia | 2020 | UEK75498.1 |  | DENV-4 | Venezuela | 2007 | ACH99664.1 |
| DENV-2 | Papua New Guinea | 2000 | QSX24591.1 |  | DENV-4 | Colombia | 2005 | ACW83013.1 |
| DENV-2 | India | 2016 | QCZ24972.2 |  | DENV-4 | USA | 1995 | ACS32037.1 |
| DENV-2 | Pakistan | 2022 | UZJ81459.1 |  | DENV-4 | Singapore | 2019 | UWM25513.1 |
