# Supplementary material for: Cross-serotype immunity elicited by a consensus dengue NS1 mRNA vaccine in mice
Source: PLoS One. 2026 Aug 6;21(8):e0355389. doi: 10.1371/journal.pone.0355389 (PMC13446656; doi:10.1371/journal.pone.0355389)
Supplement: Supplementary file 4 [file pone.0355389.s004.docx]

**S2 Table.**

| **mRNA construct** | **Z-average (nm)** | **Polydispersity index** | **Encapsulation (%)** |
| --- | --- | --- | --- |
| DENV-1 consensus | 69.29 | 0.1152 | 91.1 |
| DENV-2 consensus | 67.13 | 0.0652 | 95.0 |
| DENV-3 consensus | 81.14 | 0.2005 | 92.1 |
| DENV-4 consensus | 83.26 | 0.1901 | 92.3 |
| cNS1 | 57.13 | 0.0917 | 95.8 |
