# Supplementary material for: Cross-serotype immunity elicited by a consensus dengue NS1 mRNA vaccine in mice
Source: PLoS One. 2026 Aug 6;21(8):e0355389. doi: 10.1371/journal.pone.0355389 (PMC13446656; doi:10.1371/journal.pone.0355389)
Supplement: Supplementary file 5 [file pone.0355389.s006.docx]

**Responses to Reviewer #1:**

**1. What are the sequences of 5'UTR and 3'UTR in Figure 2? Is there a polyA tail? What is the structure of PolyA tail?**

**Response:** The mRNA constructs were generated using the pUC-ccTEV-A101 vector, which incorporates proprietary 5′ and 3′ untranslated regions (UTRs) optimized for mRNA stability and translation efficiency. As these UTRs are part of a proprietary mRNA expression platform, their nucleotide sequences cannot be disclosed. We have revised the Materials and Methods section (Line No. 143-161) to clarify that all mRNAs were co-transcriptionally capped using CleanCap® AG (3′-OMe) and contained a genetically encoded poly(A) tail consisting of 101 adenine residues. In addition, all vaccine constructs were generated using the same mRNA backbone, including identical UTRs and regulatory elements, thereby minimizing potential confounding effects associated with differences in mRNA construct design.

**2. In Figure 6, were any other cytokines detected besides IFN gamma? What's the result?**

**Response:** We thank the reviewer for this valuable comment. In the present study, cellular immune responses were evaluated solely by measuring IFN-γ production using the ELISpot assay, and other cytokines were not assessed. IFN-γ was selected as the primary endpoint because it is a well-established and widely accepted marker of antigen-specific T cell responses and is commonly used to evaluate cellular immunogenicity in preclinical vaccine studies, including mRNA vaccine platforms. As the primary objective of this study was to determine whether the consensus NS1 mRNA vaccine could induce cross-serotype cellular immune responses, the IFN-γ ELISpot assay provided an appropriate and robust readout of vaccine-induced T-cell immunity.

We agree that evaluation of additional cytokines, such as IL-2, TNF-α, IL-4, and IL-10, would provide a more comprehensive characterization of the immune response induced by the vaccine. These analyses were beyond the scope of the current study but represent important directions for future investigation. To acknowledge this limitation, we have added this point in the Discussion (Line No. 391-396).

**3. What are the results of mRNA integrity and dsRNA residue detection? I haven't seen any relevant results in the manuscript? Have other quality indicators been tested? Such as template residue, purity, etc.** *AND* **6. What are the particle size and potential of LNP after encapsulation? Have other quality indicators been tested?**

**Response:** We agree that inclusion of the mRNA quality control data would strengthen the manuscript. Therefore, we have included the results of mRNA integrity and residual double-stranded RNA (dsRNA) analyses as Supplementary Figure S2. Agarose gel electrophoresis confirmed the integrity of all in vitro–transcribed mRNA constructs, while dot blot analysis demonstrated minimal residual dsRNA following cellulose-based purification. In addition, the physicochemical characteristics of the formulated mRNA-LNPs, including particle size (Z-average), polydispersity index (PDI), and encapsulation efficiency, have been included as Supplementary Table S1. These quality control assessments confirmed the integrity and consistency of the mRNA and mRNA-LNP formulations used throughout the study. The Materials and Methods section has also been revised accordingly (Line No. 161-171).

**4. What is the ratio of various lipid components used for mRNA encapsulation?**

**Response:** We have revised the Materials and Methods section to include the molar ratio of the lipid components used for mRNA encapsulation (Line No. 165-167). The lipid mixture consisted of an ionizable lipid (3D-P-DMA), DSPC, cholesterol, and PEG2000-C-DMA at a molar ratio of 50:10:38.5:1.5, respectively.

**5. Has purification been carried out after mRNA encapsulation is completed? What is the purification method?**

**Response:** Yes. Following mRNA encapsulation, the mRNA-LNP formulations underwent a post-formulation purification step consisting of buffer exchange by dialysis to remove formulation buffer components and exchange the formulation into the final storage buffer prior to downstream characterization and biological studies. The Materials and Methods section has been revised to clarify this post-formulation purification step (Line No. 167-171).

**7. As stated by the author in the preface, the NS1mRNA vaccine mainly exerts a protective effect in mouse challenge experiments, but no relevant research has been conducted in this study.**

**Response:** We agree that the present study did not evaluate protective efficacy in a dengue virus challenge model was not evaluated. The primary objective of this study was to investigate the immunogenicity of the consensus NS1 mRNA vaccine by assessing cross-serotype humoral and cellular immune responses in mice. To avoid overstating the findings of the present study, we have also revised the manuscript title to “**Cross-Serotype Immunity Elicited by a Consensus Dengue NS1 mRNA Vaccine in Mice**” which more accurately reflects the scope of the work. In addition, we have expanded the Discussion to emphasize that future studies should evaluate the protective efficacy of the consensus NS1 mRNA vaccine in dengue virus challenge models, particularly in combination with structural antigens such as prME, to determine whether complementary humoral and NS1-directed immune responses can provide broader protection against dengue virus infection (Line No. 382-387).

**Responses to Reviewer #2:**

**1. In the design of the consensus NS1 protein sequence, the authors retrieved NS1 sequences from all four dengue virus serotypes (DENV-1–4) from the NCBI GenBank database but included only 25 representative sequences per serotype published between 1964 and 2022. Could the authors explain the rationale for selecting only 25 sequences per serotype to design this vaccine candidate? Were these sequences chosen to represent different geographical regions, genotypes, lineages, and temporal distributions? Please clarify this important point.**

**Response:** The selection of 25 NS1 sequences per dengue virus serotype was intended to generate representative serotype-specific consensus sequences while maintaining a manageable dataset for multiple sequence alignment and consensus sequence generation. The selected sequences were retrieved from the NCBI Protein database and were chosen to maximize geographic and temporal diversity, representing isolates collected from multiple countries between 1964 and 2022. Although the sequences were not specifically selected based on genotype or lineage, the inclusion of geographically and temporally diverse isolates was intended to capture representative sequence diversity within each serotype. Sequences containing incomplete coding regions, ambiguous amino acid residues, duplicated entries, or extensive gaps were excluded from the analysis. We have clarified the sequence selection criteria and consensus design strategy in the Materials and Methods section (Line No. 122-129) and provided the complete list of selected sequences, including their accession numbers, countries of origin, and year of isolation, in Supplementary Table S2.

**2. For the in vitro mRNA transfection and protein expression analyses, the authors used Western blotting to evaluate NS1 expression. However, the levels of secreted NS1 protein in the culture supernatants were not assessed. Why was an ELISA not performed to quantify NS1 secretion? Please explain the rationale for choosing Western blotting over other approaches that would allow quantitative assessment of secreted NS1.**

**Response:** We appreciate the reviewer's comment. The primary objective of the in vitro expression experiments was to confirm that the mRNA constructs successfully expressed and secreted NS1 protein, rather than to quantitatively compare secretion levels among the different constructs. Therefore, Western blot analysis was selected because it enables verification of the expected molecular weight and structural integrity of the expressed NS1 protein in culture supernatants. In this study, Western blot was intended as a qualitative method to verify NS1 expression and secretion rather, and band intensity was not interpreted as a quantitative measure of relative protein expression. We agree that ELISA would provide a quantitative assessment of secreted NS1 protein and could further characterize secretion efficiency. However, such quantitative analysis was beyond the scope of the present study, which focused primarily on evaluating the immunogenicity of the consensus NS1 mRNA vaccine.

**3. Along the same lines, following immunization with either the serotype-specific NS1 mRNA vaccines or the cNS1 mRNA vaccine, samples were collected at different time points post-immunization (days 0, 14, and 35). Were circulating NS1 protein levels measured in the vaccinated mice at any time point? This is a critical issue, as soluble NS1 has been shown to activate multiple immune pathways and contribute to flavivirus-associated pathogenesis and disease complications. Please address this point.**

**Response:** We thank the reviewer for raising this important point. We agree that circulating soluble NS1 has been implicated in the pathogenesis of dengue virus infection through its ability to modulate multiple immune pathways and disrupt endothelial barrier function.

In the present study, circulating NS1 protein levels were **not** measured following immunization. The primary objective of our work was to evaluate the immunogenicity and protective efficacy of the NS1 mRNA vaccine candidates rather than to characterize the pharmacokinetics of vaccine-derived NS1 expression in vivo. We acknowledge that assessment of circulating NS1 would provide valuable information regarding the expression profile and potential systemic exposure following vaccination.

However, it is important to note that the NS1 protein expressed following mRNA vaccination differs fundamentally from the high levels of soluble NS1 detected during active dengue virus infection. During natural infection, NS1 is continuously produced by replicating virus over several days and can accumulate in the circulation at concentrations associated with disease severity. In contrast, mRNA vaccines mediate transient antigen expression that is localized primarily to cells at the injection site and draining lymph nodes, with expression declining as the mRNA is degraded. Consequently, systemic exposure to vaccine-derived NS1 is expected to be substantially lower and of much shorter duration than that observed during dengue virus infection.

Furthermore, vaccinated mice in our study showed no clinical signs of illness, weight loss, or other adverse effects following immunization, suggesting that the vaccine was well tolerated under the experimental conditions employed. Nevertheless, we agree that direct measurement of circulating NS1 would further strengthen the safety evaluation of NS1 mRNA vaccines. We have acknowledged this as a limitation of the current study and consider it an important area for future investigation, together with detailed biodistribution and pharmacokinetic analyses of vaccine-derived NS1 expression (Line No. 388-391).

**4. In the Western blot analyses evaluating NS1 expression in transfected cells, why did the authors not use the same anti-NS1 antibody employed in the immunofluorescence assay (IFA) to detect all NS1 proteins, in addition to assessing the constructs containing the tPA and IgE signal peptides? Please clarify the rationale for this approach.**

**Response:** We apologize for any confusion caused by the original presentation. The same rabbit anti-DENV2-NS1 polyclonal antibody (Invitrogen, catalog no. PA5-32207) was used for both the immunofluorescence assay (IFA) and Western blot analyses. In the IFA, the antibody was used to evaluate the intracellular expression and localization of NS1 in transfected cells, whereas in the Western blot assay it was used to confirm the secretion of NS1 proteins in culture supernatants. The original manuscript states that the same anti-NS1 primary antibody was used for Western blot analysis; however, we appreciate the opportunity to clarify this point.

**5. Related to the previous point, there appear to be substantial differences in NS1 protein expression levels among the transfected cells. How might these differences affect the main outcomes and conclusions of the study? Please discuss this issue.**

**Response:** We appreciate the reviewer's insightful comment. Although differences in Western blot band intensity were observed among the NS1 constructs, Western blot analysis in this study was performed as a qualitative assay to confirm NS1 expression and secretion rather than to quantitatively compare protein expression levels. Therefore, band intensity should not be interpreted as a direct measure of relative protein expression.

In addition, the observed differences in band intensity may partly reflect differences in antibody recognition rather than actual expression levels. Detection was performed using a rabbit anti-DENV-2 NS1 polyclonal antibody, which may preferentially recognize epitopes that are more conserved in the DENV-2 NS1 sequence, potentially resulting in variable detection efficiency among the constructs.

Importantly, the main conclusions of this study are based on the in vivo immunogenicity results, including NS1-specific antibody responses and IFN-γ ELISpot assays, rather than on qualitative Western blot signals. This point has already been addressed in the Results section (Line No. 283-288).

**6. When evaluating anti-NS1 IgG responses in sera collected from vaccinated mice, which NS1 antigen was used to coat the ELISA plates? Was a commercially available recombinant NS1 protein used, or was the antigen derived from NS1 secreted by transfected cells in this study? Please specify and clarify this point, as it is critical for interpreting IgG reactivity and specificity.**

**Response:** We thank the reviewer for this important comment. The ELISA plates were coated with commercially recombinant dengue virus NS1 proteins representing each of the four DENV serotypes (Sino Biological, China). NS1 proteins secreted from transfected cells were not used as coating antigens in this study. We selected commercially available recombinant NS1 proteins because they provide standardized and serotype-specific antigens, enabling consistent evaluation of vaccine-induced NS1-specific IgG responses as well as cross-serotype antibody reactivity. We have revised the Materials and Methods section to further clarify the source of the ELISA coating antigens (Line No. 213-216).

**7. The same question applies to the evaluation of IgG responses induced by cNS1 immunization. Which antigen was used to coat the ELISA plates in these experiments? Please provide clarification.**

**Response:** The same ELISA procedure was used to evaluate sera from mice immunized with the consensus NS1 (cNS1) mRNA vaccine. Commercially available recombinant NS1 proteins representing each of the four dengue virus serotypes (DENV-1–4) (Sino Biological, China) were used as the coating antigens. NS1 proteins secreted from transfected cells were not used in these assays. The use of standardized recombinant NS1 proteins allowed us to consistently evaluate vaccine-induced NS1-specific IgG response against each serotype and to assess the breadth of cross-serotype antibody reactivity elicited by the cNS1 mRNA vaccine. We have clarified this information in the Materials and Methods section (Line No. 210-216).
