# Supplementary material for: Cross-serotype immunity elicited by a consensus dengue NS1 mRNA vaccine in mice
Source: PLoS One. 2026 Aug 6;21(8):e0355389. doi: 10.1371/journal.pone.0355389 (PMC13446656; doi:10.1371/journal.pone.0355389)
Supplement: Supplementary file 7 [file pone.0355389.s002.docx]

**S2 Fig**
