# Supplementary material for: Cross-serotype immunity elicited by a consensus dengue NS1 mRNA vaccine in mice
Source: PLoS One. 2026 Aug 6;21(8):e0355389. doi: 10.1371/journal.pone.0355389 (PMC13446656; doi:10.1371/journal.pone.0355389)
Supplement: Peer review history

# Peer review: Decision Letter 0

**DOI:** 10.1371/journal.pone.0355389.r001

**Authors:** Julian Ruiz-Saenz

1 Jul 2026

PONE-D-26-23191

Cross-Serotype Protective Immunity Elicited by a Consensus Dengue NS1 mRNA Vaccine in Mice

PLOS One

Dear Dr. Ketloy,

Thank you for submitting your manuscript to PLOS ONE. After careful consideration, we feel that it has merit but does not fully meet PLOS ONE’s publication criteria as it currently stands. Therefore, we invite you to submit a revised version of the manuscript that addresses the points raised during the review process.

Please submit your revised manuscript by Aug 30 2026 11:59PM. If you will need more time than this to complete your revisions, please reply to this message or contact the journal office at plosone@plos.org. When you're ready to submit your revision, log on to https://www.editorialmanager.com/pone/ and select the 'Submissions Needing Revision' folder to locate your manuscript file.

Please include the following items when submitting your revised manuscript:

- A letter that responds to each point raised by the academic editor and reviewer(s). You should upload this letter as a separate file labeled 'Response to Reviewers'.
- A marked-up copy of your manuscript that highlights changes made to the original version. You should upload this as a separate file labeled 'Revised Manuscript with Track Changes'.
- An unmarked version of your revised paper without tracked changes. You should upload this as a separate file labeled 'Manuscript'.

If you would like to make changes to your financial disclosure, please include your updated statement in your cover letter. Guidelines for resubmitting your figure files are available below the reviewer comments at the end of this letter.

If applicable, we recommend that you deposit your laboratory protocols in protocols.io to enhance the reproducibility of your results. Protocols.io assigns your protocol its own identifier (DOI) so that it can be cited independently in the future. For instructions see: https://journals.plos.org/plosone/s/submission-guidelines#loc-laboratory-protocols. Additionally, PLOS ONE offers an option for publishing peer-reviewed Lab Protocol articles, which describe protocols hosted on protocols.io. Read more information on sharing protocols at https://plos.org/protocols?utm_medium=editorial-email&utm_source=authorletters&utm_campaign=protocols.

As the corresponding author, your ORCID iD is verified in the submission system and will appear in the published article. PLOS supports the use of ORCID, and we encourage all coauthors to register for an ORCID iD and use it as well. Please encourage your coauthors to verify their ORCID iD within the submission system before final acceptance, as unverified ORCID iDs will not appear in the published article. *Only*  the individual author can complete the verification step; PLOS staff *cannot*  verify ORCID iDs on behalf of authors.

We look forward to receiving your revised manuscript.

Kind regards,

**Julian Ruiz-Saenz, PhD**

Academic Editor

PLOS One

Journal Requirements:

When submitting your revision, we need you to address these additional requirements.

1. Please ensure that your manuscript meets PLOS ONE's style requirements, including those for file naming. The PLOS ONE style templates can be found at

https://journals.plos.org/plosone/s/file?id=wjVg/PLOSOne_formatting_sample_main_body.pdf and

https://journals.plos.org/plosone/s/file?id=ba62/PLOSOne_formatting_sample_title_authors_affiliations.pdf

2. We note that the grant information you provided in the ‘Funding Information’ and ‘Financial Disclosure’ sections do not match.

When you resubmit, please ensure that you provide the correct grant numbers for the awards you received for your study in the ‘Funding Information’ section.

3. Thank you for stating the following in the below Acknowledgments Section of your manuscript: [C. Ketloy acknowledges funding from the National Vaccine Institute (NVI), under grant number 2567.1/27, and the Ratchadapiseksompotch Fund, Faculty of Medicine, Chulalongkorn University, under grant number RA-MF-26/68. We also acknowledge partial support from the UK–South East Asia Vaccine Manufacturing Research Hub for this research.]

We note that you have provided funding information that is not currently declared in your Funding Statement. However, funding information should not appear in the Acknowledgments section or other areas of your manuscript. We will only publish funding information present in the Funding Statement section of the online submission form.

Please remove any funding-related text from the manuscript and let us know how you would like to update your Funding Statement. Currently, your Funding Statement reads as follows:

Please include your amended statements within your cover letter; we will change the online submission form on your behalf.

4. In the online submission form, you indicated that [Additional raw data are available from the corresponding author upon reasonable request].

All PLOS journals now require all data underlying the findings described in their manuscript to be freely available to other researchers, either 1. In a public repository, 2. Within the manuscript itself, or 3. Uploaded as supplementary information.

This policy applies to all data except where public deposition would breach compliance with the protocol approved by your research ethics board. If your data cannot be made publicly available for ethical or legal reasons (e.g., public availability would compromise patient privacy), please explain your reasons on resubmission and your exemption request will be escalated for approval.

5. If the reviewer comments include a recommendation to cite specific previously published works, please review and evaluate these publications to determine whether they are relevant and should be cited. There is no requirement to cite these works unless the editor has indicated otherwise.

[Note: HTML markup is below. Please do not edit.]

Reviewers' comments:

Reviewer's Responses to Questions

**Comments to the Author**

1. Is the manuscript technically sound, and do the data support the conclusions?

The manuscript must describe a technically sound piece of scientific research with data that supports the conclusions. Experiments must have been conducted rigorously, with appropriate controls, replication, and sample sizes. The conclusions must be drawn appropriately based on the data presented.

Reviewer #1: Partly

Reviewer #2: Yes

**********

2. Has the statistical analysis been performed appropriately and rigorously?

Reviewer #1: I Don't Know

Reviewer #2: Yes

**********

3. Have the authors made all data underlying the findings in their manuscript fully available?

The PLOS Data policy requires authors to make all data underlying the findings described in their manuscript fully available without restriction, with rare exception (please refer to the Data Availability Statement in the manuscript PDF file). The data should be provided as part of the manuscript or its supporting information, or deposited to a public repository. For example, in addition to summary statistics, the data points behind means, medians and variance measures should be available. If there are restrictions on publicly sharing data—e.g. participant privacy or use of data from a third party—those must be specified.

Reviewer #1: Yes

Reviewer #2: Yes

**********

4. Is the manuscript presented in an intelligible fashion and written in standard English?

PLOS ONE does not copyedit accepted manuscripts, so the language in submitted articles must be clear, correct, and unambiguous. Any typographical or grammatical errors should be corrected at revision, so please note any specific errors here.

Reviewer #1: Yes

Reviewer #2: Yes

**********

5. Review Comments to the Author

Please use the space provided to explain your answers to the questions above. You may also include additional comments for the author, including concerns about dual publication, research ethics, or publication ethics. (Please upload your review as an attachment if it exceeds 20,000 characters)

**Reviewer #1:**

In this study, the author studied that Cross-Serotype Protective Immunity Elicited by a Consensus Dengue NS1 mRNA Vaccine in Mice. The manuscript has the following issues.

1. What are the sequences of 5'UTR and 3'UTR in Figure 2? Is there a polyA tail? What is the structure of polyA tail?

2. In Figure 6, were any other cytokines detected besides IFN gamma? What's the result?

3. What are the results of mRNA integrity and dsRNA residue detection? I haven't seen any relevant results in the manuscript? Have other quality indicators been tested? Such as template residue, purity, etc.

4. What is the ratio of various lipid components used for mRNA encapsulation?

5. Has purification been carried out after mRNA encapsulation is completed? What is the purification method?

6. What are the particle size and potential of LNP after encapsulation? Have other quality indicators been tested?

7. As stated by the author in the preface, the NS1mRNA vaccine mainly exerts a protective effect in mouse challenge experiments, but no relevant research has been conducted in this study.

**Reviewer #2:**

The manuscript entitled “Cross-Serotype Protective Immunity Elicited by a Consensus Dengue NS1 mRNA Vaccine in Mice” by Tharakhet and colleagues examines anti-NS1 IgG responses in NS1-immunized mice using an mRNA–lipid nanoparticle (mRNA–LNP) vaccine formulation. The authors demonstrate that a consensus NS1 (cNS1) mRNA vaccine induces cross-serotype humoral and cellular immune responses in mice, highlighting the potential of consensus antigen design to broaden immune recognition of DENV NS1. These findings suggest that NS1-directed immunity may complement conventional vaccine strategies focused on structural antigens.

Although this is a well-written and well-structured study addressing an important gap that continues to limit the effectiveness of antibody responses elicited by dengue vaccine candidates, several methodological and interpretative issues must be addressed before this manuscript can be considered for publication in PLOS ONE.

Please address the following concerns:

Major Comments

1. In the design of the consensus NS1 protein sequence, the authors retrieved NS1 sequences from all four dengue virus serotypes (DENV-1–4) from the NCBI GenBank database but included only 25 representative sequences per serotype published between 1964 and 2022. Could the authors explain the rationale for selecting only 25 sequences per serotype to design this vaccine candidate? Were these sequences chosen to represent different geographical regions, genotypes, lineages, and temporal distributions? Please clarify this important point.

2. For the in vitro mRNA transfection and protein expression analyses, the authors used Western blotting to evaluate NS1 expression. However, the levels of secreted NS1 protein in the culture supernatants were not assessed. Why was an ELISA not performed to quantify NS1 secretion? Please explain the rationale for choosing Western blotting over other approaches that would allow quantitative assessment of secreted NS1.

3. Along the same lines, following immunization with either the serotype-specific NS1 mRNA vaccines or the cNS1 mRNA vaccine, samples were collected at different time points post-immunization (days 0, 14, and 35). Were circulating NS1 protein levels measured in the vaccinated mice at any time point? This is a critical issue, as soluble NS1 has been shown to activate multiple immune pathways and contribute to flavivirus-associated pathogenesis and disease complications. Please address this point.

4. In the Western blot analyses evaluating NS1 expression in transfected cells, why did the authors not use the same anti-NS1 antibody employed in the immunofluorescence assay (IFA) to detect all NS1 proteins, in addition to assessing the constructs containing the tPA and IgE signal peptides? Please clarify the rationale for this approach.

5. Related to the previous point, there appear to be substantial differences in NS1 protein expression levels among the transfected cells. How might these differences affect the main outcomes and conclusions of the study? Please discuss this issue.

6. When evaluating anti-NS1 IgG responses in sera collected from vaccinated mice, which NS1 antigen was used to coat the ELISA plates? Was a commercially available recombinant NS1 protein used, or was the antigen derived from NS1 secreted by transfected cells in this study? Please specify and clarify this point, as it is critical for interpreting IgG reactivity and specificity.

7. The same question applies to the evaluation of IgG responses induced by cNS1 immunization. Which antigen was used to coat the ELISA plates in these experiments? Please provide clarification.

**********

6. PLOS authors have the option to publish the peer review history of their article (what does this mean?). If published, this will include your full peer review and any attached files.

If you choose “no”, your identity will remain anonymous but your review may still be made public.

**Do you want your identity to be public for this peer review?**  For information about this choice, including consent withdrawal, please see our Privacy Policy.

Reviewer #1: No

Reviewer #2: No

**********

[NOTE: If reviewer comments were submitted as an attachment file, they will be attached to this email and accessible via the submission site. Please log into your account, locate the manuscript record, and check for the action link "View Attachments". If this link does not appear, there are no attachment files.]

To ensure your figures meet our technical requirements, please review our figure guidelines: https://journals.plos.org/plosone/s/figures

You may also use PLOS’s free figure tool, NAAS, to help you prepare publication quality figures: https://journals.plos.org/plosone/s/figures#loc-tools-for-figure-preparation.

NAAS will assess whether your figures meet our technical requirements by comparing each figure against our figure specifications.

# Author comment: Author response to Decision Letter 1

**DOI:** 10.1371/journal.pone.0355389.r002

8 Jul 2026

Responses to Reviewer #1:

1. What are the sequences of 5'UTR and 3'UTR in Figure 2? Is there a polyA tail? What is the structure of PolyA tail?

Response: The mRNA constructs were generated using the pUC-ccTEV-A101 vector, which incorporates proprietary 5′ and 3′ untranslated regions (UTRs) optimized for mRNA stability and translation efficiency. As these UTRs are part of a proprietary mRNA expression platform, their nucleotide sequences cannot be disclosed. We have revised the Materials and Methods section (Line No. 143-161) to clarify that all mRNAs were co-transcriptionally capped using CleanCap® AG (3′-OMe) and contained a genetically encoded poly(A) tail consisting of 101 adenine residues. In addition, all vaccine constructs were generated using the same mRNA backbone, including identical UTRs and regulatory elements, thereby minimizing potential confounding effects associated with differences in mRNA construct design.

2. In Figure 6, were any other cytokines detected besides IFN gamma? What's the result?

Response: We thank the reviewer for this valuable comment. In the present study, cellular immune responses were evaluated solely by measuring IFN-γ production using the ELISpot assay, and other cytokines were not assessed. IFN-γ was selected as the primary endpoint because it is a well-established and widely accepted marker of antigen-specific T cell responses and is commonly used to evaluate cellular immunogenicity in preclinical vaccine studies, including mRNA vaccine platforms. As the primary objective of this study was to determine whether the consensus NS1 mRNA vaccine could induce cross-serotype cellular immune responses, the IFN-γ ELISpot assay provided an appropriate and robust readout of vaccine-induced T-cell immunity.

We agree that evaluation of additional cytokines, such as IL-2, TNF-α, IL-4, and IL-10, would provide a more comprehensive characterization of the immune response induced by the vaccine. These analyses were beyond the scope of the current study but represent important directions for future investigation. To acknowledge this limitation, we have added this point in the Discussion (Line No. 391-396).

3. What are the results of mRNA integrity and dsRNA residue detection? I haven't seen any relevant results in the manuscript? Have other quality indicators been tested? Such as template residue, purity, etc. AND 6. What are the particle size and potential of LNP after encapsulation? Have other quality indicators been tested?

Response: We agree that inclusion of the mRNA quality control data would strengthen the manuscript. Therefore, we have included the results of mRNA integrity and residual double-stranded RNA (dsRNA) analyses as Supplementary Figure S2. Agarose gel electrophoresis confirmed the integrity of all in vitro–transcribed mRNA constructs, while dot blot analysis demonstrated minimal residual dsRNA following cellulose-based purification. In addition, the physicochemical characteristics of the formulated mRNA-LNPs, including particle size (Z-average), polydispersity index (PDI), and encapsulation efficiency, have been included as Supplementary Table S1. These quality control assessments confirmed the integrity and consistency of the mRNA and mRNA-LNP formulations used throughout the study. The Materials and Methods section has also been revised accordingly (Line No. 161-171).

4. What is the ratio of various lipid components used for mRNA encapsulation?

Response: We have revised the Materials and Methods section to include the molar ratio of the lipid components used for mRNA encapsulation (Line No. 165-167). The lipid mixture consisted of an ionizable lipid (3D-P-DMA), DSPC, cholesterol, and PEG2000-C-DMA at a molar ratio of 50:10:38.5:1.5, respectively.

5. Has purification been carried out after mRNA encapsulation is completed? What is the purification method?

Response: Yes. Following mRNA encapsulation, the mRNA-LNP formulations underwent a post-formulation purification step consisting of buffer exchange by dialysis to remove formulation buffer components and exchange the formulation into the final storage buffer prior to downstream characterization and biological studies. The Materials and Methods section has been revised to clarify this post-formulation purification step (Line No. 167-171).

7. As stated by the author in the preface, the NS1mRNA vaccine mainly exerts a protective effect in mouse challenge experiments, but no relevant research has been conducted in this study.

Response: We agree that the present study did not evaluate protective efficacy in a dengue virus challenge model was not evaluated. The primary objective of this study was to investigate the immunogenicity of the consensus NS1 mRNA vaccine by assessing cross-serotype humoral and cellular immune responses in mice. To avoid overstating the findings of the present study, we have also revised the manuscript title to “Cross-Serotype Immunity Elicited by a Consensus Dengue NS1 mRNA Vaccine in Mice” which more accurately reflects the scope of the work. In addition, we have expanded the Discussion to emphasize that future studies should evaluate the protective efficacy of the consensus NS1 mRNA vaccine in dengue virus challenge models, particularly in combination with structural antigens such as prME, to determine whether complementary humoral and NS1-directed immune responses can provide broader protection against dengue virus infection (Line No. 382-387).

Responses to Reviewer #2:

1. In the design of the consensus NS1 protein sequence, the authors retrieved NS1 sequences from all four dengue virus serotypes (DENV-1–4) from the NCBI GenBank database but included only 25 representative sequences per serotype published between 1964 and 2022. Could the authors explain the rationale for selecting only 25 sequences per serotype to design this vaccine candidate? Were these sequences chosen to represent different geographical regions, genotypes, lineages, and temporal distributions? Please clarify this important point.

Response: The selection of 25 NS1 sequences per dengue virus serotype was intended to generate representative serotype-specific consensus sequences while maintaining a manageable dataset for multiple sequence alignment and consensus sequence generation. The selected sequences were retrieved from the NCBI Protein database and were chosen to maximize geographic and temporal diversity, representing isolates collected from multiple countries between 1964 and 2022. Although the sequences were not specifically selected based on genotype or lineage, the inclusion of geographically and temporally diverse isolates was intended to capture representative sequence diversity within each serotype. Sequences containing incomplete coding regions, ambiguous amino acid residues, duplicated entries, or extensive gaps were excluded from the analysis. We have clarified the sequence selection criteria and consensus design strategy in the Materials and Methods section (Line No. 122-129) and provided the complete list of selected sequences, including their accession numbers, countries of origin, and year of isolation, in Supplementary Table S2.

2. For the in vitro mRNA transfection and protein expression analyses, the authors used Western blotting to evaluate NS1 expression. However, the levels of secreted NS1 protein in the culture supernatants were not assessed. Why was an ELISA not performed to quantify NS1 secretion? Please explain the rationale for choosing Western blotting over other approaches that would allow quantitative assessment of secreted NS1.

Response: We appreciate the reviewer's comment. The primary objective of the in vitro expression experiments was to confirm that the mRNA constructs successfully expressed and secreted NS1 protein, rather than to quantitatively compare secretion levels among the different constructs. Therefore, Western blot analysis was selected because it enables verification of the expected molecular weight and structural integrity of the expressed NS1 protein in culture supernatants. In this study, Western blot was intended as a qualitative method to verify NS1 expression and secretion rather, and band intensity was not interpreted as a quantitative measure of relative protein expression. We agree that ELISA would provide a quantitative assessment of secreted NS1 protein and could further characterize secretion efficiency. However, such quantitative analysis was beyond the scope of the present study, which focused primarily on evaluating the immunogenicity of the consensus NS1 mRNA vaccine.

3. Along the same lines, following immunization with either the serotype-specific NS1 mRNA vaccines or the cNS1 mRNA vaccine, samples were collected at different time points post-immunization (days 0, 14, and 35). Were circulating NS1 protein levels measured in the vaccinated mice at any time point? This is a critical issue, as soluble NS1 has been shown to activate multiple immune pathways and contribute to flavivirus-associated pathogenesis and disease complications. Please address this point.

Response: We thank the reviewer for raising this important point. We agree that circulating soluble NS1 has been implicated in the pathogenesis of dengue virus infection through its ability to modulate multiple immune pathways and disrupt endothelial barrier function.

In the present study, circulating NS1 protein levels were not measured following immunization. The primary objective of our work was to evaluate the immunogenicity and protective efficacy of the NS1 mRNA vaccine candidates rather than to characterize the pharmacokinetics of vaccine-derived NS1 expression in vivo. We acknowledge that assessment of circulating NS1 would provide valuable information regarding the expression profile and potential systemic exposure following vaccination.

However, it is important to note that the NS1 protein expressed following mRNA vaccination differs fundamentally from the high levels of soluble NS1 detected during active dengue virus infection. During natural infection, NS1 is continuously produced by replicating virus over several days and can accumulate in the circulation at concentrations associated with disease severity. In contrast, mRNA vaccines mediate transient antigen expression that is localized primarily to cells at the injection site and draining lymph nodes, with expression declining as the mRNA is degraded. Consequently, systemic exposure to vaccine-derived NS1 is expected to be substantially lower and of much shorter duration than that observed during dengue virus infection.

Furthermore, vaccinated mice in our study showed no clinical signs of illness, weight loss, or other adverse effects following immunization, suggesting that the vaccine was well tolerated under the experimental conditions employed. Nevertheless, we agree that direct measurement of circulating NS1 would further strengthen the safety evaluation of NS1 mRNA vaccines. We have acknowledged this as a limitation of the current study and consider it an important area for future investigation, together with detailed biodistribution and pharmacokinetic analyses of vaccine-derived NS1 expression (Line No. 388-391).

4. In the Western blot analyses evaluating NS1 expression in transfected cells, why did the authors not use the same anti-NS1 antibody employed in the immunofluorescence assay (IFA) to detect all NS1 proteins, in addition to assessing the constructs containing the tPA and IgE signal peptides? Please clarify the rationale for this approach.

Response: We apologize for any confusion caused by the original presentation. The same rabbit anti-DENV2-NS1 polyclonal antibody (Invitrogen, catalog no. PA5-32207) was used for both the immunofluorescence assay (IFA) and Western blot analyses. In the IFA, the antibody was used to evaluate the intracellular expression and localization of NS1 in transfected cells, whereas in the Western blot assay it was used to confirm the secretion of NS1 proteins in culture supernatants. The original manuscript states that the same anti-NS1 primary antibody was used for Western blot analysis; however, we appreciate the opportunity to clarify this point.

5. Related to the previous point, there appear to be substantial differences in NS1 protein expression levels among the transfected cells. How might these differences affect the main outcomes and conclusions of the study? Please discuss this issue.

Response: We appreciate the reviewer's insightful comment. Although differences in Western blot band intensity were observed among the NS1 constructs, Western blot analysis in this study was performed as a qualitative assay to confirm NS1 expression and secretion rather than to quantitatively compare protein expression levels. Therefore, band intensity should not be interpreted as a direct measure of relative protein expression.

In addition, the observed differences in band intensity may partly reflect differences in antibody recognition rather than actual expression levels. Detection was performed using a rabbit anti-DENV-2 NS1 polyclonal antibody, which may preferentially recognize epitopes that are more conserved in the DENV-2 NS1 sequence, potentially resulting in variable detection efficiency among the constructs.

Importantly, the main conclusions of this study are based on the in vivo immunogenicity results, including NS1-specific antibody responses and IFN-γ ELISpot assays, rather than on qualitative Western blot signals. This point has already been addressed in the Results section (Line No. 283-288).

6. When evaluating anti-NS1 IgG responses in sera collected from vaccinated mice, which NS1 antigen was used to coat the ELISA plates? Was a commercially available recombinant NS1 protein used, or was the antigen derived from NS1 secreted by transfected cells in this study? Please specify and clarify this point, as it is critical for interpreting IgG reactivity and specificity.

Response: We thank the reviewer for this important comment. The ELISA plates were coated with commercially recombinant dengue virus NS1 proteins representing each of the four DENV serotypes (Sino Biological, China). NS1 proteins secreted from transfected cells were not used as coating antigens in this study. We selected commercially available recombinant NS1 proteins because they provide standardized and serotype-specific antigens, enabling consistent evaluation of vaccine-induced NS1-specific IgG responses as well as cross-serotype antibody reactivity. We have revised the Materials and Methods section to further clarify the source of the ELISA coating antigens (Line No. 213-216).

7. The same question applies to the evaluation of IgG responses induced by cNS1 immunization. Which antigen was used to coat the ELISA plates in these experiments? Please provide clarification.

Response: The same ELISA procedure was used to evaluate sera from mice immunized with the consensus NS1 (cNS1) mRNA vaccine. Commercially available recombinant NS1 proteins representing each of the four dengue virus serotypes (DENV-1–4) (Sino Biological, China) were used as the coating antigens. NS1 proteins secreted from transfected cells were not used in these assays. The use of standardized recombinant NS1 proteins allowed us to consistently evaluate vaccine-induced NS1-specific IgG response against each serotype and to assess the breadth of cross-serotype antibody reactivity elicited by the cNS1 mRNA vaccine. We have clarified this information in the Materials and Methods section (Line No. 210-216).

Attachment Submitted filename: Response to Reviewers.docx

# Peer review: Decision Letter 1

**DOI:** 10.1371/journal.pone.0355389.r003

**Authors:** Julian Ruiz-Saenz

22 Jul 2026

Cross-Serotype Immunity Elicited by a Consensus Dengue NS1 mRNA Vaccine in Mice

PONE-D-26-23191R1

Dear Dr. Ketloy,

We’re pleased to inform you that your manuscript has been judged scientifically suitable for publication and will be formally accepted for publication once it meets all outstanding technical requirements.

Within one week, you’ll receive an e-mail detailing the required amendments. When these have been addressed, you’ll receive a formal acceptance letter and your manuscript will be scheduled for publication.

An invoice will be generated when your article is formally accepted. Please note, if your institution has a publishing partnership with PLOS and your article meets the relevant criteria, all or part of your publication costs will be covered. Please make sure your user information is up-to-date by logging into Editorial Manager at Editorial Manager® and clicking the ‘Update My Information' link at the top of the page. For questions related to billing, please contact billing support.

If your institution or institutions have a press office, please notify them about your upcoming paper to help maximize its impact. If they’ll be preparing press materials, please inform our press team as soon as possible -- no later than 48 hours after receiving the formal acceptance. Your manuscript will remain under strict press embargo until 2 pm Eastern Time on the date of publication. For more information, please contact onepress@plos.org.

Kind regards,

Julian Ruiz-Saenz, PhD

Academic Editor

PLOS One

Additional Editor Comments (optional):

Reviewers' comments:

Reviewer's Responses to Questions

**Comments to the Author**

1. If the authors have adequately addressed your comments raised in a previous round of review and you feel that this manuscript is now acceptable for publication, you may indicate that here to bypass the “Comments to the Author” section, enter your conflict of interest statement in the “Confidential to Editor” section, and submit your "Accept" recommendation.

Reviewer #1: All comments have been addressed

Reviewer #2: All comments have been addressed

**********

2. Is the manuscript technically sound, and do the data support the conclusions?

The manuscript must describe a technically sound piece of scientific research with data that supports the conclusions. Experiments must have been conducted rigorously, with appropriate controls, replication, and sample sizes. The conclusions must be drawn appropriately based on the data presented.

Reviewer #1: Yes

Reviewer #2: (No Response)

**********

3. Has the statistical analysis been performed appropriately and rigorously?

Reviewer #1: Yes

Reviewer #2: (No Response)

**********

4. Have the authors made all data underlying the findings in their manuscript fully available?

The PLOS Data policy requires authors to make all data underlying the findings described in their manuscript fully available without restriction, with rare exception (please refer to the Data Availability Statement in the manuscript PDF file). The data should be provided as part of the manuscript or its supporting information, or deposited to a public repository. For example, in addition to summary statistics, the data points behind means, medians and variance measures should be available. If there are restrictions on publicly sharing data—e.g. participant privacy or use of data from a third party—those must be specified.

Reviewer #1: Yes

Reviewer #2: (No Response)

**********

5. Is the manuscript presented in an intelligible fashion and written in standard English?

PLOS ONE does not copyedit accepted manuscripts, so the language in submitted articles must be clear, correct, and unambiguous. Any typographical or grammatical errors should be corrected at revision, so please note any specific errors here.

Reviewer #1: Yes

Reviewer #2: (No Response)

**********

6. Review Comments to the Author

Please use the space provided to explain your answers to the questions above. You may also include additional comments for the author, including concerns about dual publication, research ethics, or publication ethics. (Please upload your review as an attachment if it exceeds 20,000 characters)

Reviewer #1: The author has basically responded to the reviewer's questions. This manuscript can be acceptable for publication now.

Reviewer #2: All comments have been adequately addressed; therefore, no further comments are raised from this reviewer.

**********

7. PLOS authors have the option to publish the peer review history of their article (what does this mean?). If published, this will include your full peer review and any attached files.

If you choose “no”, your identity will remain anonymous but your review may still be made public.

**Do you want your identity to be public for this peer review?**  For information about this choice, including consent withdrawal, please see our Privacy Policy.

Reviewer #1: No

Reviewer #2: No

**********

# Editor report: Acceptance letter

**DOI:** 10.1371/journal.pone.0355389.r004

**Authors:** Julian Ruiz-Saenz

PONE-D-26-23191R1

PLOS One

Dear Dr. Ketloy,

I'm pleased to inform you that your manuscript has been deemed suitable for publication in PLOS One. Congratulations! Your manuscript is now being handed over to our production team.

At this stage, our production department will prepare your paper for publication. This includes ensuring the following:

* All references, tables, and figures are properly cited

* All relevant supporting information is included in the manuscript submission,

* There are no issues that prevent the paper from being properly typeset

You will receive further instructions from the production team, including instructions on how to review your proof when it is ready. Please keep in mind that we are working through a large volume of accepted articles, so please give us a few days to review your paper and let you know the next and final steps.

Lastly, if your institution or institutions have a press office, please let them know about your upcoming paper now to help maximize its impact. If they'll be preparing press materials, please inform our press team within the next 48 hours. Your manuscript will remain under strict press embargo until 2 pm Eastern Time on the date of publication. For more information, please contact onepress@plos.org.

You will receive an invoice from PLOS for your publication fee after your manuscript has reached the completed accept phase. If you receive an email requesting payment before acceptance or for any other service, this may be a phishing scheme. Learn how to identify phishing emails and protect your accounts at https://explore.plos.org/phishing.

If we can help with anything else, please email us at customercare@plos.org.

Thank you for submitting your work to PLOS ONE and supporting open access.

Kind regards,

PLOS ONE Editorial Office Staff

on behalf of

Dr. Julian Ruiz-Saenz

Academic Editor

PLOS One
